# The Power of the Political in an Urbanizing International

**DOI:** 10.1177/0304375418762048

**Published:** 2018-04-02

**Authors:** Jodok Troy

**Affiliations:** 1 Department of Political Science, University of Innsbruck, Innsbruck, Austria; 2 The Europe Center, Stanford University, Stanford, CA, USA (visiting scholar 2016–2018)

**Keywords:** urbanization, the political, political theory, Hans J. Morgenthau, conflict, cities, power

## Abstract

In this article, I argue that there is a startling resonance between Hans Morgenthau’s conception of the political and power and recent analyses of an urbanizing international realm. By making this connection clear, I depart from a mechanistic understanding of politics, which tends to inform both conventional International Relations views and some claims in urban studies pertaining to the rise of global cities as international actors. Turning to Morgenthau’s conception of the political and power also has wider implications for International Relations studies of urbanization: it helps explain a tendency toward depoliticization caused by ignoring the conflictual character of the political. The emphasis on the political, on the other hand, serves as a bridge between International Relations and urbanization studies by creating conditions for the repoliticization of urban space. After illustrating the existential manifestation of the political and its violent outfalls, the remainder of this article turns to its relational and dialogical manifestation that points out the shortcomings of reading the political merely as an existential concept in the context of urbanization.

Post-structuralists and critical international political theorists have long lamented the depoliticization of international politics. Depoliticization, critics contend, is the result of policies seeking mechanical causes of political problems in order to apply appropriate “instruments” to solve them in terms of both political practice and political analysis.^1^ This is particularly the case where the fields of urban politics and international politics overlap. Indeed, a thriving research branch of International Relations contends with *the politics* of urbanization, but there is modest research on how urbanization affects *the political*. While there is an abundance of analysis about the politics of an urbanizing international realm, international studies often overlook the ramifications of “the political” such as the political’s permeating empirical and normative features of politics and power.

The political is different from an understanding of politics that is narrowly framed as a set of practices and institutions.^2^ But what makes a matter a political one? To answer this question, I rely on the twentieth-century Realist Hans J. Morgenthau who defines the political as a site in which contests of power are involved. This conceptualization assumes that a persistent antagonism of human conduct pervades politics without being fixed on any one principle such as assumptions of an evil human nature.^3^ What makes Morgenthau’s conceptualization of the political so appealing is its empirical and normative conceptualization. Morgenthau’s conceptualization is equally equipped to provide insights into processes of politization and depoliticization, while at the same time providing a normative backing of any political conception.^4^ Morgenthau has been one of the most vocal critics of depoliticizing tendencies in the practice and study of politics, criticising the “faith in the perfectibility of human society through reason and a brash optimism that all problems were susceptible to technical solution.”^5^ Because of his critique of technical solutions to genuine political problems, Morgenthau’s concept is particularly germane for contemporary policies and research in an urbanizing international realm.

In this article, I argue that Morgenthau’s conception of the political departs from a mechanistic understanding of politics that informs both conventional International Relations views on urbanization and some of the claims of urban studies pertaining to the rise of global cities as international actors. The shapes and ramifications of the political in an urban international realm show a puzzling pattern: urbanization propels the resurgence of existential and dialogical forms of the political, where policies politicize and depoliticize the urban space. Urbanization increases the density of human agglomerations, potentially accelerating rivalry and conflict that is characteristic of an existential form of the political. In urban space, powerlessness of human agglomerations likely becomes more complex and discharges into new forms of the political.

By illustrating the different avenues the political can wield in processes of urbanization, the article points out that desire and imitation among human beings are fundamental features of the political. Attention to human’s imitation of the desire of others shows that it is not a definite human trait or any fixed ground of principal that leads to different manifestations of the political. Rather, the different manifestations of the political are the results of unconscious human interaction. Urbanization requires a thorough analysis of their political micro practices in the international realm. They affect politics in organizing social life and pose challenges on conceptualizing the political. Attention “to the city” is thus, as Warren Magnusson recalls, “therapeutic because it draws attention to the micro-practices that enable urban life, which are inherently political.”^6^

In facing today’s challenges of urbanization, Morgenthau’s conceptions of the political and power help us better understand depoliticization tendencies in urban policies and international relations as a whole.^7^ Classical Realism has always been a warning voice for the epistemological and ontological pitfalls of converting “the world into a rationalist utopia, instead of engaging with the world as it is,”^8^ and for ruling out the autonomous sphere of the political in order to distinguish between political and nonpolitical facts.^9^ In particular, Morgenthau’s conceptions of the political can serve as a bridge between International Relations scholars and urbanization scholars as to how the urban, the political, and the international relate to one another. Urbanization does not only produce depoliticization and conflict, but it also creates the conditions for the politicization of urban spaces.

I first delineate macrotheoretical assumptions of urbanization within the international realm. This illustrates the way that urbanization generates outputs immanent in and external to the international system. Such outputs make the case that the politics of urbanization, such as technologization, call attention to different forms of the political. By turning to conceptions of the political as outlined by Morgenthau, the next step contributes to the burgeoning literature on urbanization, showing how conventional analytical approaches rest on notions of depoliticization. After demonstrating the existential and conflictual manifestation of the political, the remainder of this article turns to the dialogical manifestations of the political. It points to the shortcomings of existential readings of the political in the context of an urbanizing international realm.

## Urbanization, Cities, and International Studies

Questions of how urbanization affects international politics have developed into prosperous research in international studies. Some states, for example, no longer have the monopoly of violence in urban spaces they used to have, urban warfare challenges international security, and global cities engage in diplomacy, a practice dominated by states.^10^ Urbanization today has different impacts on state stability and often goes hand in hand with dissident foreign policies of cities. Cities’ takes on diplomacy likely affect the international system by adding new actors to the international stage.^11^ Yet cities have always been a nucleus of state formation, and they have developed their own foreign policies and attempts of diplomatic outreach. Charles Tilly, for example, illustrated this at the entanglement of early modern wealthy urban European elites, capital cities, and strongmen.^12^ Even more so today, urbanizing global politics are confronted with more actors participating in diplomacy.^13^ In other words, urbanization produces new entities that rival the state.^14^

Since the publication of Sassen’s *The Global City*,^15^ scholars of global politics have turned their attention to urbanization writ large. Even more so, ever since arguing that the entanglement of globalization and localization—“glocalization”^16^—is the defining feature of global change, scholars have increasingly noted that urbanization does not only feature domestic change. Rather, processes of urbanization challenge the traditional variables of the international system. Benjamin Barber’s praise of pragmatic mayors in an age of dysfunctional nation-states is the latest contribution to this trend, pointing out city agency in the international realm.^17^ Yet, as I illustrate in the next section, it is not only the state system as the traditional focus of International Relation studies that is affected by urbanization.

For multilevel governance and liberal theories, city networks are a dependent or an intervening variable in the framework of global governance as global cities wield extraordinary economic influence in the international realm.^18^ For critical theories, cities exemplify the urbanization of warfare and the violent impact of uncontrolled urbanization spread by unequal economic growth.^19^ Although there is “little evidence that networks of cities can take on the ‘anarchical’ society of states,”^20^ actual city regions matter in a virtualized world.^21^ The physical appearance of powers such as China is characterized by an urban revolution accompanied by the securitization of infrastructure.^22^ What is more, their appearance illustrates that city cooperation contributes to great power cooperation.^23^ For constructivists, cities are norm entrepreneurs.^24^ Cities have been more successful than several states in carrying out sustainable environmental policies. What is more, capitals are the “urban core” of diplomatic missions,^25^ incarnating the *genius loci* of diplomatic theory and displaying yet another strand of cities as norm entrepreneurs. Cities also have been at the forefront of conflicts in the international environment—whether as strategic objectives in war or as sites of showdowns in urban warfare. In short, urban space is a “crucial component of war and central to human conflict.”^26^ Intracity violence generates an internationalized threat, and cities are prime sites of conflict and its resolution.^27^ “Fragile” and “failing” cities are at the center of attention in matters of international security, just as the nation-state was before.^28^ While urban areas may be resilient zones of coexistence in some instances, they are just that: zones of coexisting entities, existing next to each other and breeding fertile grounds for conflict as I point out below.

Historical singularities of cities and urbanization must not be conceptually confused with the city. The claim that urbanization generates cities and is characterized by an increased density among the population resembles only one aspect of urbanization. Urbanization might as well also come with a decrease in density following peri-urbanization (i.e., urban growth that creates hybrid landscapes). Here, I focus on the broad trend of urbanization’s production of density. My emphasis on urbanization does not imply that I neglect the importance of rural movements. Moreover, I do not claim that urbanization is primarily a metropolitan dynamic. Such a notion is challenged by urban research, which puts forward a notion of an “urban society”^29^ that might be more akin to recognizing the various forms of the political. However, even studies critical of the theoretical and empirical conceptions of the “urban age” conclude that urbanization is a global phenomenon.^30^ At no time before have more people lived together in this many conglomerations, constituting a rising level of human interconnectedness.^31^ The US National Intelligence Council’s Report characterizes the “world as urban” and identifies urbanization as part of the mega trend of changing demographic patterns.^32^ Whereas urbanization in the nineteenth century was largely peaceful,^33^ recent urbanization, because of its scale and speed, forbids a direct comparison to its preceding instances.

Processes and outcomes of urbanization are not limited to the practices and institutions associated with international politics illustrated above. Those processes and outcomes also account for what critical scholars refer to as depoliticizing effects. They affect the various competing antagonistic dimensions of the political. Yet the space or lack of space that is characteristic of urbanization does not become political only by virtue of existing power rivalries or competing interests.^34^ Understanding what makes urbanization a driving force for the different shapes of the political thus needs further investigation.

## The Political in an Urbanizing World

Other than Carl Schmitt’s infamous concept of the political,^35^ Morgenthau’s concept of the political is one that, first, rests on a relational approach but, second, does not get rid of the antagonistic feature that permeates international politics.^36^ Morgenthau’s conceptualization frames the political as a space of public deliberation. Whether or not a matter becomes political “depends on circumstances of time and place and does not result from a ground of principle.”^37^ The political, Morgenthau holds, is a “quality,” which “lies in the degree of intensity of the connections between the object of the state’s activity and the state.”^38^ In doing so, Morgenthau rejects foundational principles such as assuming an evil human nature. In outlining this conception of the political, to the confusion of International Relations theorists, Morgenthau meanders between Nietzschean skepticism and Weberian ideal types when conceptualizing the political and power.^39^ Morgenthau contends that a contest for the will to power turns a matter political either by maintaining such a will, increasing it, or demonstrating it.^40^ In a rudimentary attempt to explain this is the case, Morgenthau pointed out the human trait of comparison.^41^ In this empirical concept of power, Morgenthau stressed this trait as the desire for power that “manifests itself as the desire to maintain the range of one’s own person with regard to others, to increase it, or to demonstrate it.” At its root, the individual’s desire for power “concerns itself” with the “position among his fellows.”^42^

Morgenthau’s concepts of the political and power cannot be reduced to a specific intellectual relationship. Rather, he understands them as psychogenic and intersubjective conditions. Morgenthau conceptualized power empirically (maintaining, increasing, or demonstrating it) but also normatively.^43^ Based on the conception of the political as a result and quality of human interaction, Morgenthau leaned toward the notion of power as something existing between them. This notion of power is similar to that of Hannah Arendt whose work stimulated Morgenthau’s. Arendt defined power as a product of action arising between people. Therefore, only a group can possess power. Once the group breaks down, so does power. Arendt, similar to Morgenthau, opposes the Aristotelian notion of the inherently political human being. One person alone can never be political, let alone, as Morgenthau stresses, that humans are not only “political” beings but consist of a composite nature including biological, ethical, religious, and other traits.^44^

Power, on the other side, is ultimately driven by the psychogenic and intersubjective human trait to prove oneself, an essential characteristic of the political.^45^ This trait of comparison, to prove oneself, is prone to discharge into rivalry, a human condition of which classical Realists were well aware.^46^ Desire and imitation of others longings are “intervening variables” of the political. They are relational human traits, as desire “resides not in any one object or person by itself, but rather is constituted in the relationships between people.”^47^ Yet desire is imitative, hence it does not assume individual human beings as sovereign and autonomous with the capacity and authority to judge and decide before they act as commonly assumed.^48^

To illustrate Morgenthau’s epistemological move toward examining the human desire that concerns itself with the “position among his fellows,” it is useful to briefly turn to mimetic theory. Mimetic theory, as formulated by René Girard, holds that humans are characterized by mutual imitation. Human conduct is not about patterns of behavior but about the imitation of desire. In Girardian mimetic theory, the Greek term “mimesis” links imitation and desire. Put differently, humans are characterized by borrowed desire. As long as the desired good remains nonexclusive (e.g., the love of parents for their children), mimesis produces positive outcomes. Once those goods are exclusive (e.g., social positions), mimetic rivalry has the potential to wind up in violence. For mimetic theory, the “principal source of violence between human beings is mimetic rivalry, the rivalry resulting from imitation of a model who becomes a rival or of a rival who becomes a model.”^49^ Urbanization increases the likeness of rivalry over the same goods since there are more models available, which are capable of becoming a potential rival. Reduction in distance between the desired object, the imitator, and the imitated increases the likeness of conflict because the imitator sees the imitated as an obstacle on the way toward the desired object.

This analytic approach to the political opens up an alternative framework for thinking about urbanization in the international realm. Most conceptions of the global city and urbanization as a complex web of networks assume a relationship between cause and effect among different entities, which allows them to escape the political character of urbanization. As Tedesco has proposed in this journal, the relational feature of global cities can be “approached not in terms of competing ontologies but as a multitude of logics through which co-constitutive interactions can potentially be governed…as modes of governing and self-governing, these logics operate as political logics, a recognition that is missing in the relational ontologies of the global city and even of urbanization.”^50^ The following section reveals two of these political logics by looking at different contentions of the political in light of Morgenthau’s conceptualization.

### The Existential Political: Urbanization and Violence

Empirical research shows that population growth, which results in increased density in urban space, can increase the likeliness of conflict,^51^ thus potentially becoming a driving force of the political’s existential logic. This is not to say that there is a causal link between urban density and violence.^52^ Nonetheless, the very existence of the potential of the political’s existential logic illustrates that the density accompanying urbanization exceeds authorities and residents responding to it.^53^ “Density,” according to McFarlane, is at “once a topographical” problem and a “problem of topological politics of space.”^54^ Urban uprisings are increasing on a global scale.^55^ The “Arab Spring,” for example, has been partly motivated by the access of youth to social media, which made them aware of Western lifestyles in other parts of the world, buying into a “justice-based international order.”^56^ Urbanization, in this regard, serves as an equalizing force that makes people aware of what others have. As such, urbanization contributes in various ways to the generalization of the international realm, where complexity and diversity are acknowledged and dealt with politically.^57^

Finding and defining identity is more difficult where there is less difference between people, such as in dense urban areas. Since the end of the Cold War, cities have made the coexistence of many diverse identities possible.^58^ But, as Pierre Bourdieu observed, “Social identity lies in difference, and difference is asserted against what is closest, which represents the greatest threat.”^59^ The point here is not that identities have to clash to emerge and shape in a violent manner.^60^ Rather, the point is that the vanishing of differences may lead to greater potential for future conflict facing a growing globalization.^61^ In terms of mimetic theory, this means that it is not difference but sameness that is the problem in political interaction. Rivalry indeed emerges from “relative” rather than absolute “disadvantage.”^62^ Social, political, and economic inequalities do not lead, per se, to violence. But given the increasing possibilities of comparison between individuals, there is certainly a potential danger of the outbreak of violence. Mimetic theory, as introduced above, points to the danger of comparison and Morgenthau’s concept of the political assumed the individual’s struggle for the “position among his fellows”^63^ as a condition for the contestation over power, which characterizes the political.

In a world that becomes “flat”^64^ and where physical distance is less of an issue, humans tend to compare their lifestyles with others and imitate the material and nonmaterial desire of others. The democratic dogma of egalitarianism potentially pushes rivalry to its edges, based on wholesaling the possibility to imitate the desire of others.^65^ Globalization’s homogenization efforts led to a globalized “Jihad” on the one side and a globalized “McWorld” on the other side, both propelled by the globalization of resentment.^66^ Peer group comparison, an awareness of what others have and want, is one reason why growing economic wealth, pressed by globalization, goes hand in hand with strong sentiments of nationalism.^67^ The more options there are to imitate, the more likely it is that people struggle over the options available. Urbanization, seen from the angle of demographic development and leading to partial improvement of social and economic conditions for some, raises the stakes of rivalry over relative gains for others.

Research on revolutions reinforces these theoretical claims. Revolutions are more likely to occur after the experience of improved living conditions. In other words, it is the relative deprivation of basic living conditions that drives the “gap between what people feel rightfully entitled to and what they are capable of achieving under existing circumstances.”^68^ Sooner or later the long-fueled outbreak of desiring what others desire, often disguised as nationalism, serves as a condition of civic conflict in the urban sphere. For example, research on political conflict in sub-Saharan and Asian cities points out that urban social disorder is associated with low economic growth and hybrid democratic regimes rather than levels of development or inequality.^69^ An early study on urbanization and world politics concluded that most worries about political order and social well-being in the context of “rapid urban growth and underemployment [are] *political* rather than economic.”^70^

Civic conflict is “directly related to the urban realm in that it generally takes place *in* cities and it is linked to the socioeconomic and spatial particularities of cities.”^71^ While civil conflict is “essentially instrumental, civic conflict is generally expressive and…falls short of taking control of formal structures of power.”^72^ In other words, security becomes urbanized.^73^ Sassen describes this expressive character of civic conflict as the complexity of powerlessness.^74^ The protest movements during the “Arab Spring” and other urban uprisings may have had no power in a material sense but they still made politics via their presence on city streets and squares. One reason why civic conflict is globally on the rise is that “civic conflict is a common *response* to that rapid urbanisation.”^75^

Violence and disorder within groups, which tend to outnumber violence between groups, are persistent components of political conduct. Paradoxically, however, scholars of political theory and international studies became concerned with a notion of the political that aligned with a desire for order, despite the fact that disorder and violence remain the offspring of political entities.^76^ As James Scott concludes in his study of the state, the “enlightenment belief in the self-improvement of men became, by degrees, a belief in the perfectibility of social order.”^77^ The most apparent example of this belief in the self-improvement of men can be seen in city planning that fails to account for the subjectivity of those living there.^78^ This is obvious, for example, in the dull condition of the suburbs in the northern hemisphere, planned at the drawing board and being illustrative for a mechanistic urban policy. The Banlieus of Paris are only the most prominent examples, eventually declared to be “counter excavations”^79^ in the search for nonpolitical instruments to solve political urban problems.

Urbanization in the global North set in after there was a consolidated state in place, one that did not promote civic conflict because it could not provide the essential needs, security, and welfare for its citizens. Cities in the global North began to rise before the communication revolution, which made the rest of the world aware of Western materialism.^80^ What is more, the urban space as an economic hub once tended to solve conflicts by “economizing” them via material trade-offs. Today, this intervening economizing action of the urban is challenged around the globe. Cities no longer only mitigate conflict. It is more likely that structural challenges of civic and civil order make cities breed conflict themselves.^81^ The urbanizing international realm, however, also illustrates the relational carvings of the political, showing that the political signifies only the possibility, not necessity of violence as it will be outlined in the reminder of the article.

### The Relational Political: The Power of the Political on the Global Street

Urbanization compels people as an existential driving force of the political. However, urbanization also drives the logic of the political, which seeks to safeguard a space outside politics understood as conflict and violence. This is a place where irenic politics of deliberation and consent can grow. As outlined above, other than Schmitt’s existential conception of the political, Morgenthau’s conception of the political focused on its dialogical and relational aspect. Morgenthau forestalled Sassen’s concept of the “global street”^82^ as one where the political is in the making. This concept illustrates that cities enable powerless human agglomerations in the urban sphere to become complex, representing new forms of power and politics that can take conflicting as well as irenic routes.^83^ Events on the “global street” from the Tahrir Square in Cairo, the Maidan in Kiev to Western suburbs illustrate the potential of civic conflict as outlined above. Yet the “global street” is different from the classical street and plaza as an (European) ritualized ordered public space and activity. Whereas boulevards and piazzas symbolized rituals, the street and the square represent action and space for divergent interests and rivalries over them. This development enables “the powerless: urban space makes their powerlessness complex, and in that complexity rests the possibility of making the political, making the civic”^84^—a conception of the political very much like Morgenthau’s normative one. Yet, as illustrated before, this development potentially also increases the danger of existential and violent capabilities of the political.

The political is a matter of degree, of how potentially violent conflict can get. Morgenthau arrived at a seemingly narrow conception of the political with his definition of the autonomy of the political sphere and the concept of “interest defined in terms of power.”^85^ However, as he cautions, the nature of the political “depends on circumstances of time and place and does not result from a ground of principle.”^86^ Urbanization causes one of the “confrontations between divergent wills, interest, and the forms of power they can wield.”^87^ This is not to equate the conflictual character of the political with violence. Neither Schmitt nor Morgenthau did so. The political signifies only the possibility not the necessity of violence. At this point, critical scholars of urbanization arrived at the same conclusion as Morgenthau in his seemingly narrow concept of the political. Morgenthau’s normative approach addresses the centrality of power in politics “without reducing politics to an undifferentiated sphere of violence, to distinguish legitimate forms of political power, to insulate the political sphere from physical violence, and to discern the social structures that such a strategy requires to be successful.”^88^ As soon as conflicts turn violent, the political character in the sense of Morgenthau’s conceptualization ceases to exist. Not surprisingly, some scholars suggest a keen relationship between Morgenthau (and classical Realism more generally) and critical theory,^89^ making his concepts even more apt to deal with the problems of urbanization as they are pointed out by critical theorists.

A matter becomes political when, in interpersonal relationships, “the drive to prove oneself takes an explicit interest in humans”^90^ This is the ground of the tragic character of human existence. The tragedy of human existence is a “result of an excessiveness of the drive to prove oneself; the potential gain of pleasure and the objects to which that gain is directed are without limit.”^91^ Given the intersubjective impetus of power, for Morgenthau, power does not rest on individuals. Rather, it “signifies the consent of people to temporarily come together in collective speech and action, in order to create institutions, laws, and norms.”^92^ Therefore, interpersonal relationships, not individuals, drive political power. This is how Morgenthau’s conceptions of power and the political demonstrate why and how “powerless” urban agglomerates can form politics by developing normative productive power even while they are still in constant danger of transforming into violence.

Morgenthau’s concept of the political bears analytical potential for an urbanizing international scene because it does not reduce urban politics to “authoritative decision-making at a smaller scale than national units,”^93^ which reduces politics to decision-making.^94^ The powerless urban agglomerates strive to manifest power expressed in domestic revolutions, starting on the street and eventually discharging in civic and civil conflict and war but in any case with unforeseeable outcomes. The powerless ones on the global streets are also able to exercise political power because there is a difference between military and political power that rests on a psychological relationship. In “the exercise of physical violence,” Morgenthau cautioned, “the psychological element of the political relationship is lost.”^95^ Stressing this caution, he opposed “depoliticized forms of power that restrict the human creative capacity for building meaningful spatio-temporal articulations of the common good.”^96^ Powerless urban agglomerates are a form of politics, illustrating the importance of spaces and actual places where the political is in the making,^97^ but they also carry their them own violent temptations with.

In the complexity of powerlessness, civic conflict resembles a political bottom-up phenomenon that can develop a degree of political intensity as the defining marker of the political. Conflict and power are “pervasive facet[s] of human existence,” which illustrates the analytical value of Morgenthau’s empirical and normative conceptualization of the political and power. Interstate conflict remained exceptional chiefly because it typically constituted a particularly intense and thus explosive form of antagonism. At this point, we “most commonly encounter what Schmitt described as potentially violent conflicts between friend and foe.”^98^ The backburner of this conception of the political is its criticism of the modern “escape from power” and depoliticization. The “escape from power” hinders recognition of the politicizing potential of civil conflict in urban spaces. The political, in Morgenthau’s terms, is created through human interaction via dialogue. As such, it has no limits.^99^ The Western modernist attempt to create a neutral sphere and the rationalization of postpolitical space as the means to “escape power,” caused a focus on the expected outcome (Westernization and stabilization) of modernization, at the expense of all other observed trends.^100^ Eventually, this phenomenon reinforced the prevailing conception of the political as a distinction between friend and enemy, blindsided on other possible carvings of the political. One of the paradoxes of “the urban age” is that many of its associated processes resemble an attempt to rationalize a postpolitical space. The “urban age” gives way to existential as well as relational carvings of the political.

## Conclusion

This article illustrated that urbanization does not only lead to desirable outcomes such as city agency. Rather, urbanization is also prone to foster civic conflict and existential capabilities of the political and politics. By examining the potential outcomes of civic conflict, the article contributes to the literature concerning the “social physics of the city”^101^ under the lens of the political and power. The notion of the “city as a system” slowly takes control of the political, often leading to depoliticization in the politics of urbanization. As large parts of societies around the globe increasingly become alike, this phenomenon is even more apparent with the spread of urbanized populations.^102^ This is particularly the case when the state lacks the power to control its territory and when inadequate adoptions of political order eventually lead to the urban reclaiming of the political, for example, via civic conflict or informal activism. Urbanization in Gaza City, Mogadishu, and their peers, for example, takes place simultaneously with state building and state failure.

Based on the existential and relational conceptions of the political and the empirical and normative conceptions of power constituting it, this article argued that scholars of international studies need to take both concepts seriously, as both are propelled by an urbanizing world. Merely focusing on depoliticization markers, such as the lack of institutional power of the state, the anarchical structure of the international system, or the agency of cities, neglects the importance of informal violence in civic conflict. The article’s second major point is that a relational conception of the political helps us understand the new forms of power that the political wields in the processes of urbanization. Powerless urban agglomerates potentially constitute forms of politics in spaces and actual places where the political is in the making amid depoliticizing effects. Morgenthau’s concept of the political, where contests of power are involved, helps to empirically explore the impacts of an urbanizing international realm. Specifically, his approach gives us a conceptual lens for examining the conflictual and violent outgrowths or urbanization, while it at the same time provides a theoretical invigoration of the political in the making on the “global street.”

The struggle over different manifestations and carvings of the political, in the form of demonstrations claiming city squares, civic conflict, or organized violence, is a deeply human affair that will not go away. In order to examine its complications, civic conflicts in urban centers need empirical analysis as well as normative theoretical contention. A significant part of overcoming the dynamic of human rivalry based on different conceptualizations of the political starts with recognizing this dynamic of human rivalry in the international realm. Recognizing this dynamic, however, requires a resistance to any foundational ideological, social, or economical principle that drives the manifestation of the political.

## Notes

1. Jenny Edkins, *Poststructuralism and International Relations: Bringing the Political Back in* (Boulder, CO: Lynne Rienner, 1999); Robert W. Cox, “Social Forces, States and World Orders: Beyond International Relations Theory,”
*Millennium: Journal of International Studies* 10, no. 2 (1981). doi:10.1177/03058298810100020501. On the notion of “instruments” see, for example, Robert O. Keohane, “Big Questions in the Study of World Politics,” in *The Oxford Handbook of International Relations*, ed. Christian Reus-Smit and Duncan Snidal (Oxford, UK: Oxford University Press, 2010), 708–15.

2. Michi Ebata and Beverly Neufeld, *Confronting the Political in International Relations* (New York: Macmillan Press, 2000). On the distinction between a narrow and a broader meaning of the political see Edkins, *Poststructuralism and International Relations*, 2; Chantal Mouffe, *On the Political* (London, UK: Routledge, 2006).

3. Hans J. Morgenthau, Behr Hartmut, and Rösch, Felix, *The Concept of the Political* (New York: Palgrave Macmillan, 2012); Vassilios Paipais, “Between Politics and the Political: Reading Hans J. Morgenthau’s Double Critique of Depoliticiastion,” *Millennium: Journal of International Studies* 42, no. 2 (2013): 367.

4. Felix Rösch, “Pouvoir, Puissance, and Politics: Hans Morgenthau’s Dualistic Concept of Power?” *Review of International Studies* 40 (2014). doi: 10.1017/S0260210513000065.

5. Patrick T. Jackson, “Hans Morgenthau’s Pilgrimage Among the Engineers,” in *Progressivism and US Foreign Policy between the World Wars*, ed. Molly Cochran and Cornelia Navari (New York: Palgrave Macmillan, 2017), 297; Hans J.Morgenthau, *Scientific Man Vs. Power Politics* (Chicago, IL: University of Chicago Press, 1946).

6. Warren Magnusson, “Bringing Politics Back In,” *International Political Sociology* 9, no. 1 (2015): 92. doi: 10.1111/ips.12079; Warren Magnusson, “Social Movements and the Global City,” *Millennium* 23, no. 3 (1994): 621–45; Chadwick F. Alger, “The World Relations of Cities: Closing the Gap between Social Science Paradigms and Everyday Human Experience,” *International Studies Quarterly* 34, no. 4 (1990): 493–518.

7. Delacey Tedesco, “The Urbanization of Politics: Relational Ontologies or Aporetic Practices?” *Alternatives: Global, Local, Political* 37, no. 4 (2012); Erik Swyngedouw, “Unlocking the Mind-trap: Politicising Urban Theory and Practice,” *Urban Studies* 54, no. 1 (2017). doi: 10.1177/0042098016671475; Julie-Anne Boudreau, *Global Urban Politics: Informalization of the State* (Malden, MA: Polity Press, 2017), 331–47.

8. Jackson, “Hans Morgenthau’s Pilgrimage among the Engineers,” 297.

9. See most paradigmatically, Morgenthau, *Scientific Man*, 1946.

10. Michele Acuto, *Global Cities, Governance and Diplomacy: The Urban Link* (Abingdon, UK: Routledge, 2013); Mark Abrahamson, *Global Cities* (New York: Oxford University Press, 2004); Simon Curtis, “Global Cities and the Transformation of the International System,” *Review of International Studies* 37, no. 4 (2011); Saskia Sassen, “Cities in Today’s Global Age,” *SAIS Review* 29 (2009): 3–32; Noah J. Toly, “Governing Global Urbanization: From Technics to Politics,” *International Studies Review* 15, no. 4 (2013). doi: 10.1111/misr.12078; Simon Curtis, *The Power of Cities in International Relations* (New York: Routledge, 2016); Michael Mark Amen, *Cities and Global Governance: New Sites for International Relations* (Farnham, UK: Ashgate, 2011); Stephen Graham, ed., *Cities, War, and Terrorism: Towards an Urban Geopolitics* (Malden, MA: Blackwell, 2004); Martin Coward, *Urbicide: The Politics of Urban Destruction* (London, UK: Routledge, 2009), 1923–47.

11. Abrahamson, *Global Cities*, 2004; Acuto, *Global Cities, Governance and Diplomacy*, 2013; Amen, *Cities and Global Governance*, 2011; Curtis, “Global Cities and the Transformation,” 2011; Simon Curtis, “Cities and Global Governance: State Failure or a New Global Order?” *Millennium: Journal of International Studies* 44, no. 3 (2016). doi:10.1177/0305829816637233; Frauke Kraas, “Megacities and Global Change: Key Priorities,” *The Geographical Journal* 173, no. 1 (2007). doi: 10.1111/j.1475-4959.2007.232_2.x.

12. Charles Tilly and Willem Pieter Blockmans, *Cities and the Rise of States in Europe, A.D. 1000 to 1800* (Boulder, CO: Westview Press, 1994).

13. Rogier der van Pluijm and Jan Melissen, *City Diplomacy: The Expanding Role of Cities in International Politics*, Clingendael Diplomacy Papers 10 (The Hague, the Netherlands: Netherlands Institute of International Relations “Clingendael”, 2007); Acuto, *Global Cities, Governance and Diplomacy*, 2013; Alger, “The World Relations of Cities,” 1990.

14. Jefferey M. Sellers, “Re-placing the Nation: An Agenda for Comparative Urban Politics,” *Urban Affairs Review* 40, no. 4 (2005). doi: 10.1177/1078087404272673; Andrés Cadena, Richard Dobbs, and Jaana Remes, “The Growing Economic Power of Cities,” *Journal of International Affairs* 65, no. 2 (2012): 1–17.

15. Saskia Sassen, *The Global City: New York, London, Tokyo* (Princeton, NJ: Princeton University Press, 1991).

16. Erik Swyngedouw, “Globalisation or ‘Glocalisation’? Networks, Territories and Rescaling,” *Cambridge Review of International Affairs* 17, no. 1 (2004). doi: 10.1080/0955757042000203632.

17. Benjamin R. Barber, *If Mayors Ruled the World: Dysfunctional Nations, Rising Cities* (New Haven, CT: Yale University Press, 2013); Michele Acuto, “Global Cities: Gorillas in Our Midst,” *Alternatives: Global, Local, Political* 35, no. 4 (2010): 425–48; Curtis, “Global Cities and the Transformation,” 2011; Neil Brenner, “Global Cities, Glocal States: Global City Formation and State Territorial Restructuring in Contemporary Europe,” *Review of International Political Economy* 5, no. 1 (1998). doi: 10.1080/096922998347633; Michele Acuto, “City Leadership in Global Governance,” *Global Governance* 19 (2013): 481–98.

18. UN System Task Team on the Post-2015 UN Development Agenda, “Sustainable Urbanization” (Thematic Think Piece—UN Habitat, 2012), https://goo.gl/clDHyc; Yishai Blank, “Federalism, Subsidiarity, and the Role of Local Governments in an Age of Global Multilevel Governance,” *Fordham Urban Law Journal* 37, no. 2 (2009): 509–58; Z. Neal, “Differentiating Centrality and Power in the World City Network,” *Urban Studies* 48, no. 13 (2011). doi: 10.1177/0042098010388954; Walter J. Nicholls, “Power and Governance: Metropolitan Governance in France,” *Urban Studies* 42, no. 4 (2005). doi: 10.1080/00420980500060426; Noah Toly, “Brexit, Global Cities, and the Future of World Order,” *Globalizations* 14, no. 1 (2016). doi: 10.1080/14747731.2016.1233679.

19. Saskia Sassen, “When the City Itself Becomes a Technology of War,” *Theory, Culture & Society* 27, no. 6 (2010): 33–50; Beyond the violence convex, global cities are actors in initiatives such as the “C40Cities: Climate Leadership Group” or the UCLG (“The Global Network of Cities, Local and Regional Governments”) illustrate while addressing climate change and sustainable development.

20. Christian M. Brütsch, “From Sovereign Prerogatives to Metropolitan Rule? The Anarchical Society in the Urban Age,” *International Studies Perspectives* 14, no. 3 (2013): 308. doi: 10.1111/j.1528-3585.2012.00496.x.

21. Mario Polèse, *The Wealth and Poverty of Regions: Why Cities Matter* (Chicago, IL: University of Chicago Press, 2011).

22. Martin Coward, “Hot Spots/Cold Spots: Infrastructural Politics in the Urban Age,” *International Political Sociology* 9, no. 1 (2015): 97. doi: 10.1111/ips.12081; Thomas J. Campanella, *The Concrete Dragon: China’s Urban Revolution and what it means for the World* (Princeton, NJ: Princeton Architectural Press, 2011).

23. Pengfei Ni, “Cities as the New Engine for Sino-Indian Cooperation,” *Journal of International Affairs* 64, no. 2 (2011): 143–54.

24. Brütsch, “From Sovereign Prerogatives to Metropolitan Rule?” 312.

25. Ibid., 310.

26. B. Stanley, “City Wars or Cities of Peace: (Re)Integrating the Urban into Conflict Resolution,” *GaWC Research Bulletin* 123 (2003), accessed February 26, 2018, http://www.lboro.ac.uk/gawc/rb/rb123.html. Cities have always been a “domain reflecting spatial strategies.” Michael Shapiro and Deane Neubauer, “Spatiality and Policy Discourse: Reading the Global City,” *Alternatives: Global, Local, Political* XIV (1989): 301.

27. P. H. Liotta and James F. Miskel, “The ‘Mega-Eights’: Urban Leviathans and International Instability,” *Orbis* 53, no. 4 (2009): 648. doi: 10.1016/j.orbis.2009.07.003; Graham, *Cities, War, and Terrorism*, 2004; Stephen Graham, *Cities, War, and Terrorism: Towards an Urban Geopolitics* (Malden, MA: Blackwell, 2006); Stephen Graham, *Cities under Siege: The New Military Urbanism* (London, UK: Verso, 2011); David Kilcullen, *Out of the Mountains: The Coming Age of the Urban Guerrilla* (Oxford, UK: Oxford University Press, 2013); Jon Coaffee, Paul O’Hare, and Marian Hawkesworth, “The Visibility of (In)security: The Aesthetics of Planning Urban Defences Against Terrorism,” *Security Dialogue* 40, no. 4–5 (2009): 27. doi: 10.1177/0967010609343299.

28. Richard J. Norton, “Feral Cities,” *Naval War College Review* LVI, no. 4 (2003); Ahmed Kanna, “Urban Praxis and the Arab Spring,” *City* 16, no. 3 (2012): 97–106. doi: 10.1080/13604813.2012.687879.

29. Neil Brenner and Christian Schmid, “The ‘Urban Age’ in Question,” *International Journal of Urban and Regional Research* 38, no. 3 (2014). doi: 10.1111/1468-2427.12115. See also the postcolonial critique of the “global cities research”. Jennifer Robinson, “Global and World Cities: A View from Off the Map,” *International Journal of Urban and Regional Research* 26, no. 3 (2002). doi: 10.1111/1468-2427.00397; Michiel van Meeteren, Ben Derudder, and David Bassens, “Can the Straw Man Speak? An Engagement with Postcolonial Critiques of ‘Global Cities Research’,” *Dialogues in Human Geography* 6, no. 3 (2016). doi: 10.1177/2043820616675984. I this article I do not substantively engage with the literature that animated the discipline of urban studies revolving around splintering urbanism, dual cities, or similar research strings.

30. Brenner and Schmid, “The ‘Urban Age’ in Question,” 751; Andy Merrifield, *Dialectical Urbanism: Social Struggles in the Capitalist City* (New York: Monthly Review Press, 2002); Andy Merrifield, *The New Urban Question* (London, UK: Pluto Press, 2014); Erik Swyngedouw, *Designing the Post-political City and the Insurgent Polis* (London, UK: Bedford, 2011); Warren Magnusson, *Politics of Urbanism: Seeing Like a City* (London, UK: Routledge, 2012); David Harvey, *Rebel Cities: From the Right to the City to the Urban Revolution* (London, UK: Verso, 2013).

31. Andrew Linklater, “Global Civilizing Processes and the Ambiguities of Human Interconnectedness,” *European Journal of International Relations* 16, no. 2 (2010): 158; Edward L. Glaeser, *Triumph of the City: How our Greatest Invention Makes us Richer, Smarter, Greener, Healthier, and Happier* (New York: Penguin Books, 2012); Paul L. Knox and Peter J. Taylor, eds., *World Cities in a World-system* (Cambridge, UK: Cambridge University Press, 1995).

32. National Intelligence Council, *Global trends 2030: Alternative worlds a publication of the National Intelligence Council* (Washington, DC: National Intelligence Council, 2012), accessed February 26, 2018, https://goo.gl/SizdU8, 27–30.

33. Adna Ferrin Weber, *The Growth of Cities in the Ninteenth Century: A Study in Statistics* (Ithaca, NY: Cornell University Press, 1967); Logie Barrow, Susan Finding, and François Poirier, eds., *Keeping the Lid on: Urban Eruptions and Social Control since the 19th Century* (Newcastle, UK: Cambridge Scholars, 2010).

34. Mustafa Dike, “Space, Politics, and the Political,” *Environment and Planning D: Society and Space* 23, no. 2 (2005). doi: 10.1068/d364t.

35. Schmitt conceptualized the political as one that delineates a space of power, interest, and conflict. “The specific political distinction to which political actions and motives can be reduced,” according to Schmitt, “is that between friend and enemy.” Carl Schmitt, *The Concept of the Political*, Expanded ed. (Chicago, IL: University of Chicago Press, 2007), 26. The enemy is the “other, the stranger,” 27. This particular existential conceptualization leaves aside other (violent) forms of the political such as asymmetric and guerrilla warfare, and so on. Jason Ralph, “War as an Institution of International Hierachy: Carl Schmitt’s Theory of the Partisan and Contemporary US Practice,” *Millennium—Journal of International Studies* 39, no. 2 (2010); Carl Schmitt and G. L. Ulmen, *Theory of the Partisan: Intermediate Commentary on the Concept of the Political* (New York: Telos Press, 2007); Peter U. Hohendahl, “Revolutionary War and Absolute Enemy: Rereading Schmitt’s Theory of the Partisan,” *Constellations* 18, no. 4 (2011). doi: 10.1111/j.1467-8675.2011.00657.x.

36. Paipais, “Between Politics and the Political,” 362.

37. Morgenthau, *The Concept of the Political*, 99.

38. Ibid., 102; 101 (originally in emphasizes). William E. Scheuerman, *Hans Morgenthau: Realism and Beyond* (Cambridge, UK: Polity, 2009), 33. The last two decades have seen several attempts capturing Schmitt’s and Morgenthau’s relationship; a debate that I do not join here.

39. Paipais, “Between Politics and the Political,” 362; Hans-Karl Pichler, “The Godfathers of ‘Truth’: Max Weber and Carl Schmitt in Morgenthau’s Theory of Power Politics,” *Review of International Studies* 24, no. 2 (1998): 185–200; Ido Oren, “The Unrealism of Contemporary Realism: The Tension between Realist Theory and Realists’ Practice,” *Perspectives on Politics* 7, no. 2 (2009): 283-301.

40. Morgenthau, *The Concept of the Political*, 106; Hans J. Morgenthau and Kenneth W. Thompson, *Politics among Nations: The Struggle for Power and Peace* (New York: McGraw-Hill, 1993), 50.

41. Hans J. Morgenthau, “The Evil of Politics and the Ethics of Evil,” *Ethics* 56, no. 1 (1945): 13; see also Carl Schmitt, *Ex Captivitate Salus: Erfahrungen aus der Zeit 1945/47* (Köln, Germany: Greven Verlag, 1950), 89–90.

42. Morgenthau, “The Evil of Politics and the Ethics of Evil,” 13.

43. Rösch, “Pouvoir, Puissance, and Politics,” 351.

44. Hannah Arendt, *The Human Condition*. 2nd ed., repr. (Chicago, IL: University of Chicago Press, 2006); Hans J. Morgenthau, *Politics among Nations: The Struggle for Power and Peace*, 2nd ed. (New York: Alfred A. Knopf, 1956), 12. On the similarities between Morgenthau and Arendt see Felix Rösch, “Realism as Social Criticism: The Thinking Partnership of Hannah Arendt and Hans Morgenthau,” *International Politics*, no. 50 (2013); Douglas Klusmeyer, “Beyond Tragedy: Hannah Arendt and Hans Morgenthau on Responsibility, Evil and Political Ethics,” *International Studies Review* 50, no. 6 (2009): 815–29.

45. Morgenthau, *The Concept of the Political*, 49; Morgenthau, “The Evil of Politics and the Ethics of Evil,” 13.

46. Jodok Troy, “Desire for Power or the Power of Desire? Mimetic Theory and the Heart of Twentieth-century Realism,” *Journal of International Political Theory* 11, no. 1 (2015). doi: 10.1177/1755088214555456.

47. Chris Fleming, “Mimesis, Violence, and the Sacred: An Overview of the Thought of René Girard,” in *Violence, Desire, and the Sacred: René Girard and Sacrifice in Life, Love, and Literature*, ed. Scott Cowdell, Fleming Chris, and Joel Hodge (London, UK: Bloomsbury, 2014), 2.

48. Tedesco, “The Urbanization of Politics,” 335.

49. René Girard, *I See Satan Fall like Lightning*, trans James G. Williams (Maryknoll, NY: Orbis Books, 2001), 11.

50. Tedesco, “The Urbanization of Politics,” 332.

51. J. Tir and P. F. Diehl, “Demographic Pressure and Interstate Conflict: Linking Population Growth and Density to Militarized Disputes and Wars, 1930–89,” *Journal of Peace Research* 35, no. 3 (1998). doi: 10.1177/0022343398035003004.

52. Robert Muggah, “Researching the Urban Dilemma: Urbanization, Poverty and Violence,” International Development Research Centre, https://goo.gl/yTQaEf; A. Hasan, “Can Urban Density be Made to Work for Everyone? Exploring Options for Karachi’s Low- and Lower-middle-class Settlements,” *Environment and Urbanization* 22, no. 1 (2010). doi: 10.1177/0956247810364116O. Jutersonke, R. Muggah, and D. Rodgers, “Gangs, Urban Violence, and Security Interventions in Central America,” *Security Dialogue* 40, no. 4–5 (2009). doi: 10.1177/0967010609343298; Alexandre Marc and Alys M. Willman, *Violence in the City: Understanding and Supporting Community Responses to Urban Violence* (Washington, DC: World Bank, 2010).

53. Natalie Brender, “Researching the Urban Dilemma: Urbanization, Poverty and Violence,” accessed February 26, 2018, https://goo.gl/L6RXES, 7. Unrest and riots in suburbs are also a probable indicator for Samuel Huntington’s thesis of a too-slow adjustment of political order toward changing societies, placing emphasis on factors such as urbanization rather than economic change. Samuel P. Huntington, *Political Order in Changing Societies* (New Haven CT: Yale University Press, 1968); Clifford Deaton, “The Revolution Will Not Be Occupied: Theorizing Urban Revolutionary Movements in Tehran, Prague, and Paris,” *Territory, Politics, Governance* 3, no. 2 (2015). doi: 10.1080/21622671.2014.945473; Mustafa Dike, “Disruptive Politics,” *Urban Studies* 54, no. 1 (2017). doi: 10.1177/0042098016671476.

54. Colin McFarlane, “The Geographies of Urban Density,” *Progress in Human Geography* 40, no. 5 (2016): 644. doi: 10.1177/0309132515608694. “If key sociospatial categories have been the foci of this politics—slum, suburb, skyscraper, city centre, the socially mixed city—new techniques and developments such as those around new urbanism, digital urbanism, and activist occupation have both shifted how these are understood and forced new questions about the future of density in and between cities. And yet, the political conceptions and uses of density have often been in the background of urban analysis.” Ibid. See also Ash Amin and Nigel Thrift, *Seeing Like a City* (Cambridge, UK: Polity Press, 2016).

55. Mustafa Dikec, *Urban Rage: The Revolt of the Excluded* (New Haven, CT: Yale University Press, 2018).

56. T. A. Jacoby, “A Theory of Victimhood: Politics, Conflict and the Construction of Victim-based Identity,” *Millennium—Journal of International Studies* 43, no. 2 (2015): 521; Robert Forsyth Worth, *A Rage for Order: The Middle East in Turmoil, from Tahrir Square to ISIS* (New York: Farrar, Straus and Giroux, 2016).

57. Jenny Edkins and Maja Zehfuss, “Generalising the International,” *Review of International Studies* 31, no. 3 (2005): 471.

58. Coward, *Urbicide*, 128.

59. Pierre Bourdieu, *Distinction: A Social Critique of the Judgement of Taste*, trans. Richard Nice (London: Routledge, 1984), 479. Sigmund Freud called this the “Narcissism of minor differences.” Anton Blok, “The Narcissism of Minor Differences,” *European Journal of Social Theory* 1, no. 1 (1998): 33–56.

60. I am thankful that one reviewer pointed out the importance to clarify this point.

61. Paul Laurent and Gilles Paquet, “Intercultural Relations: A Myrdal—Tocqueville—Girard Interpretative Scheme,” *International Political Science Review* 12, no. 3 (1991): 171–83.

62. Ted R. Gurr, *Why Men Rebel* (Princeton, NJ: Princeton University Press, 1970); Karl Otto Hondrich, *Wieder Krieg* (Frankfurt, Germany: Suhrkamp, 2002), 55. See also Hans Magnus Enzensberger, *Aussichten auf den Bürgerkrieg* (Frankfurt am Main, Germany: Suhrkamp, 1993), 47–48.

63. Morgenthau, “The Evil of Politics and the Ethics of Evil,” 13.

64. Thomas L. Friedman, *The World is Flat: The Globalized World in the Twenty-First Century*, Updated and expanded. (London, UK: Penguin Books, 2006).

65. Laurent and Paquet, “Intercultural Relations,” 1991.

66. Benjamin R. Barber, *Jihad vs. McWorld* (New York: Times Books, 1995); Elisabetta Brighi, “The Globalisation of Resentment: Failure, Denial and Violence in World Politics,” *Millennium—Journal of International Studies* 44, no. 3 (2016); Pankaj Mishra, *Age of Anger: A History of the Present* (New York: Farrar Straus and Giroux, 2017), 411–32.

67. Fareed Zakaria, *The Post-American World: Release 2.0*, Updated and expanded (New York: W.W. Norton, 2011), 35.

68. Jacoby, “A Theory of Victimhood,” 521. See also Gurr, *Why Men Rebel*, 1970.

69. Henrik Urdal and Kristian Hoelscher, “Explaining Urban Social Disorder and Violence: An Empirical Study of Event Data from Asian and Sub-Saharan African Cities,” *International Interactions* 38, no. 4 (2012). doi: 10.1080/03050629.2012.697427.

70. Joan Nelson, “The Urban Poor: Disruption or Political Integration in Third World Cities?” *World Politics* 22, no. 3 (1970): 395; Clionadh Raleigh, “Urban Violence Patterns Across African States,” *International Studies Review* 17, no. 1 (2015). doi: 10.1111/misr.12206.

71. J. Beall, T. Goodfellow, and D. Rodgers, “Cities and Conflict in Fragile States in the Developing World,” *Urban Studies* 50, no. 15 (2013): 8. doi: 10.1177/0042098013487775.

72. Ibid., 5.

73. Martin Coward, “Network-centric Violence, Critical Infrastructure and the Urbanization of Security,” *Security Dialogue* 40, no. 4–5 (2009). doi: 10.1177/0967010609342879.

74. Saskia Sassen, “The Global Street: Making the Political,” *Globalizations* 8, no. 5 (2011): 574. doi:10.1080/14747731.2011.622458.

75. Beall, Goodfellow, and Rodgers, “Cities and Conflict in Fragile States,” 11, italics in original.

76. Charles Tilly, “War Making and State Making as Organized Crime,” in *Bringing the State Back In*, ed. Peter B. Evans, Dietrich Rueschemeyer, and Theda Skocpol (Cambridge, UK: Cambridge University Press, 1985). Enzensberger even holds that violence is an elementary part of human life and civil war the “primary form of war.” Hans Magnus Enzensberger, *Civil War* (London, UK: Granta Books in Association With Penguin Books, 1994), 11.

77. James C. Scott, *Seeing like a State: How Certain Schemes to Improve the Human Condition Have Failed* (New Haven, CT: Yale University Press, 1998), 92.

78. Scott, *Seeing like a State*, 144; Doug Saunders, *Arrival City: The Final Migration and our Next World* (Toronto, Canada: Knopf Canada, 2010); Mike Davis, *Planet of Slums* (London, UK: Verso, 2006); Ricky Burdett, “Designing Urban Democracy: Mapping Scales of Urban Identity,” *Public Culture* 25, no. 2 (2013). doi: 10.1215/08992363-2020638.

79. Marc Angélil and Cary Siress, “The Paris Banlieue: Peripheries of Inequity,” *Journal of International Affairs* 65, no. 2 (Spring/Summer, 2012): 57–67; Mélina Germes and Georg Glasze, “Die Banlieus als Gegenorte der République: Eine Diskursanalyse neuer Sicherheitspolitiken in den Vorstädten Frankreichs,” *Geographica Helvetica* 65, no. 3 (2010): 217–28. Not terrorism, “but what has been done under the mantel of counter-terrorism, has had a significant effect on cities.” Peter Marcuse, “The ‘War on Terrorism’ and Life in Cities after September 11, 2001,” in *Cities, War, and Terrorism: Towards an Urban Geopolitics*, ed. Stephen Graham (Malden, MA: Blackwell, 2004), 263–75.

80. Liotta and Miskel, “The ‘Mega-Eights’,” 648–49.

81. Saskia Sassen, “Urban Capabilities,” *Journal of International Affairs* 65, no. 2 (2012): 85–95.

82. Sassen, “The Global Street,” 2011.

83. Paipais, “Between Politics and the Political,” 367.

84. Sassen, “The Global Street,” 575.

85. Hans J. Morgenthau, *Politics among Nations: The Struggle for Power and Peace*, 5th ed. (New York: Alfred A. Knopf, 1978), 5.

86. Morgenthau, *The Concept of the Political*, 99.

87. Michael C. Williams, “Why Ideas Matter in International Relations: Hans Morgenthau, Classical Realism, and the Moral Construction of Power Politics,” *International Organization* 58, no. 4 (2004): 644. doi: 10.1017/S0020818304040202.

88. Williams, “Why Ideas Matter in International Relations,” 637; Morgenthau, *Politics among Nations*, 31. On Morgenthau’s genealogy of power see in particular Rösch, “Pouvoir, Puissance, and Politics,” 2014.

89. See, for example, the contributions in the special issue “Interlocuting Classical Realism and Critical Theory: Negotiating ‘Divides’ in International Relations Theory,” special issue, *Journal of International Political Theory* 13, no. 1 (2017). For a different perspective, see Daniel J. Levine, “Why Hans Morgenthau Was Not a Critical Theorist (and Why Contemporary IR Realists Should Care),” *International Relations* 27, no. 1 (2013): 95–118.

90. Rösch, “Pouvoir, Puissance, and Politics,” 353.

91. Ibid., 353–54.

92. Ibid., 362.

93. Karen Mossberger, Susan E. Clarke, and Peter John, “Studying Politics in an Urban World: Research Traditions and New Directions,” in *The Oxford Handbook of Urban Politics*, ed. Peter John, Karen Mossberger, and Susan E. Clarke (Oxford, UK: Oxford University Press, 2012), 4.

94. Mustafa Dike and Erik Swyngedouw, “Theorizing the Politicizing City,” *International Journal of Urban and Regional Research* 9 (2017): 6. doi: 10.1111/1468-2427.12388.

95. Morgenthau, *Politics Among Nations*, 31.

96. Vassilios Paipais, “Between Politics and the Political, 2013.

97. Erik Swyngedouw, “Where is The Political? Insurgent Mobilisations and The Incipient Return of The Political?” *Space and Polity* 18, no. 2 (2014). doi: 10.1080/13562576.2013.879774; Dike and Swyngedouw, “Theorizing The Politicizing City,” 2017.

98. Scheuerman, *Hans Morgenthau*, 34.

99. Felix Rösch, “The Human Condition of Politics: Considering the Legacy of Hans J. Morgenthau for International Relations,” *Journal of International Political Theory* 9, no. 1 (2013): 7. doi: 10.3366/jipt.2013.0041. All of this is not to speak of Morgenthau’s writings on existential loneliness and their relevance for the modern human (urban) condition. Hans J. Morgenthau, “Love and Power,” *Commentary* 33, no. 3 (1962): 247–251.

100. Morgenthau, *Scientific Man*, 1946.

101. Sassen, “The Global Street,” 576. The city, in this sense, resembles an organism, a biological system, prone to die once shut down. Kilcullen, *Out of the Mountains*, 41.

102. Abrahamson, *Global Cities*, 15.

